# Comparison of Hi-C-Based Scaffolding Tools on Plant Genomes

**DOI:** 10.3390/genes14122147

**Published:** 2023-11-27

**Authors:** Yuze Hou, Li Wang, Weihua Pan

**Affiliations:** 1 College of Computer Science and Technology, Taiyuan University of Technology, Taiyuan 030024, China; wsdsghou@163.com; 2 Shenzhen Branch, Guangdong Laboratory of Lingnan Modern Agriculture, Genome Analysis Laboratory of the Ministry of Agriculture and Rural Affairs, Agricultural Genomics Institute at Shenzhen, Chinese Academy of Agricultural Sciences, Shenzhen 518120, China

**Keywords:** de novo assembly, scaffolding tools, scaffolding completeness, scaffolding accuracy, Hi-C

## Abstract

De novo genome assembly holds paramount significance in the field of genomics. Scaffolding, as a pivotal component within the genome assembly process, is instrumental in determining the orientation and arrangement of contigs, ultimately facilitating the generation of a chromosome-level assembly. Scaffolding is contingent on supplementary linkage information, including paired-end reads, bionano, physical mapping, genetic mapping, and Hi-C (an abbreviation for High-throughput Chromosome Conformation Capture). In recent years, Hi-C has emerged as the predominant source of linkage information in scaffolding, attributed to its capacity to offer long-range signals, leading to the development of numerous Hi-C-based scaffolding tools. However, to the best of our knowledge, there has been a paucity of comprehensive studies assessing and comparing the efficacy of these tools. In order to address this gap, we meticulously selected six tools, namely LACHESIS, pin_hic, YaHS, SALSA2, 3d-DNA, and ALLHiC, and conducted a comparative analysis of their performance across haploid, diploid, and polyploid genomes. This endeavor has yielded valuable insights in advancing the field of genome scaffolding research.

## 1. Introduction

De novo genome assembly plays a pivotal role in genomics, particularly for species lacking an established reference genome. It imparts critical insights into aspects such as genome structure, size, composition, and potential gene functions. This holds particular significance within the domains of ecology, evolutionary biology, and biodiversity research. De novo genome assembly comprises two fundamental stages: contig assembly and scaffold assembly. Contig assembly pertains to the process of organizing the initial sequencing data into abbreviated continuous sequences commonly denoted as contigs. After the generation of contigs, the subsequent phase involves their further assembly into more extensive constructs referred to as scaffolds. Scaffolds are formed via the determination of the relative positions and order of contigs. This can be accomplished by utilizing diverse sequencing techniques to facilitate the linkage between contigs, resulting in the generation of larger, more comprehensive sequences. Scaffolding leverages a variety of linkage information types to arrange and orient contigs into scaffolds. Common forms of linkage information encompass paired-end reads, bionano, physical mapping, genetic mapping, and Hi-C data. The selection of the linkage information to employ is contingent on the accessible data, the intricacy of the genome, and the precise objectives of the assembly project. In recent years, Hi-C has risen in prominence as the prevailing choice for linkage information in the scaffolding process.

Hi-C technology, initially developed for the investigation of the three-dimensional chromosomal structure and spatial interactions within genomes, encompasses a sequence of procedural stages involving cross-linking, cleavage, ligation, and sequencing. These steps enable the identification and characterization of physical interactions between various genomic regions. Due to the extensive long-range linkage information that Hi-C offers, it has found widespread utility in addressing the challenge of genome assembly scaffolding. In particular, Hi-C data adhere to two fundamental regulations. Firstly, the Hi-C signal strength between distinct chromosomes is notably lower when compared to interactions within the same chromosome. Secondly, within a chromosome, the Hi-C signal is more robust between contigs that are in close physical proximity than between contigs that are spatially distant. The former regulation supports the grouping of contigs, while the latter contributes to the accurate arrangement of contigs within each group.

At present, there exist various scaffolding tools that leverage Hi-C data, with LACHESIS [1] being the pioneering tool in utilizing Hi-C for scaffolding. LACHESIS comprises the following primary stages: Hi-C Read Alignment, Contig Grouping, and Intra-group Orientation and Sorting. LACHESIS does exhibit certain limitations, notably the necessity for users to predetermine the number of chromosomes prior to its execution. Furthermore, it lacks the capability to rectify errors within contigs in cases where such errors are present in the initial assembly. Conversely, 3d-DNA [2] tackles the error correction challenge by utilizing Hi-C reads to refine the provided contigs prior to executing the clustering, sorting, and orientation steps. SALSA [3] adopts a computational strategy in its approach. Following alignment and error correction, it builds a graph in which edge weights are determined by Hi-C links and contig lengths. These tools play an indispensable role in harnessing Hi-C data for genome scaffolding, ultimately enhancing the coherence and precision of genome assemblies. SALSA2 [4] is an extension of SALSA and introduces a hybrid graph that amalgamates information from two distinct sources: ambiguous edges derived from the GFA (Graphical Fragment Assembly) and edges acquired from Hi-C reads. This hybrid approach aims to bolster the scaffolding process by harnessing the strengths of both data types. The pin_hic [5] tool employs the N-BEST neighbor principle based on contigs and integrates data from the Hi-C contact matrix to establish a graph. This approach strives to establish connections between contigs by taking into account the top N contig neighbors. YaHS [6] adopts a distinct method by cleaving contigs at essential breakpoints, producing a contact matrix, and subsequently constructing a graph using this information. Following requisite refinements and adjustments, YaHS produces the ultimate scaffolded outcomes.

While numerous Hi-C-based scaffolding tools are available, there has been a lack of comprehensive studies evaluating their performance and offering recommendations to users. To mitigate this limitation, we undertook an evaluation of scaffolding capabilities by employing six software tools: LACHESIS, pin_hic, YaHS, SALSA2, 3d-DNA, and ALLHiC [7]. Our dataset encompassed both simulated data and authentic data sources. The genuine data originated from HiFi sequencing of a diploid strawberry genome [8], along with its associated Hi-C data. Furthermore, to bolster the robustness of our assessments, we incorporated two sets of simulated data. These simulations covered HiFi data for both a haploid genome and a tetraploid genome. In our preliminary assembly, we employed the hifiasm [9] assembly tool. The primary objective of our research is to offer valuable insights into the judicious choice of scaffolding strategies in the context of genomes with various ploidies. This guidance is designed to facilitate the generation of chromosome-level genomes characterized by heightened quality.

## 2. Materials and Methods

### 2.1. Reads and Hi-C Simulation

We had a total of three sets of raw data, all in HiFi format, comprising one set of authentic data obtained from the diploid strawberry genome and two sets of simulated data. In the case of simulated data, we produced one set to simulate a haploid genome and another to simulate a tetraploid genome. All simulated reads were generated using Pbsim3 [10], with the rice genome serving as the reference. The corresponding Hi-C signals for these simulations were generated using sim3C [11].

Among these datasets, we conducted distinct processing on the Hi-C reads generated by sim3C, as they displayed noticeable noise. Consequently, we applied filtering to the simulated Hi-C data. Although the filtering led to the elimination of certain Hi-C signals, it notably diminished the noise in the Hi-C data, thus alleviating its influence on subsequent experiments.

### 2.2. Sequence Assembly and Hi-C Mapping

We employed hifiasm to perform the initial assembly for all three datasets, producing the requisite contigs for subsequent scaffolding experiments. Notably, the Hi-C data were not integrated into this initial assembly. The preliminary assembly with hifiasm was executed using the software’s default parameters.

The subsequent step entailed mapping the Hi-C reads to the contigs generated in the preliminary assembly. For this task, we utilized the BWA [12] aligner, applying its default parameters throughout the entire process.

### 2.3. Preparation and Run

Before running these software tools, several preparatory steps were required, such as converting the alignment files in SAM format (.sam) to the binary BAM format (.bam) and generating *.bed files. Furthermore, we created an index for the contig.fasta file using samtools [13]. This index encompasses vital details about each sequence in the original FASTA file, including sequence names, sequence lengths, and the positions of sequences within the file. Indexing significantly improves the time efficiency of accessing the FASTA file. Additionally, for LACHESIS, a distinct configuration file, usually in the *.INI format, was required to be prepared. Once all of these preparatory steps were concluded, the final phase entailed executing these six software tools.

### 2.4. Evaluation of Scaffolding Tools’ Performance

Genome assembly completeness pertains to the extent to which the sequences generated during the genome assembly process faithfully and comprehensively depict the entire genome of the target organism. A complete genome assembly should encompass all chromosomes, genes, non-coding regions, and other indispensable genomic structures and elements inherent to the organism. To evaluate the integrity and accuracy of the final assembly results, we employed various metrics rooted in unique k-mers, as defined in [14]. These metrics encompass the Complete Rate (CR), the average proportion of the largest category (PLC), and the average distance difference (ADF). The Complete Rate (CR) quantifies the extent to which the final assembly aligns with the reference genome. The assessment of correctness encompasses two key aspects. Firstly, it evaluates whether the contigs corresponding to each chromosome are correctly phased. Secondly, it scrutinizes the relative arrangement of contigs within each chromosome, appraising the accuracy of their sequence linkage. Collectively, these metrics offer a comprehensive assessment of the soundness and precision of the final genome assembly outcomes.

## 3. Results

We acquired corresponding experimental results from three sets of experiments, and the analysis of these results is as follows:

### 3.1. Haploid Genome

**Figure 1:**
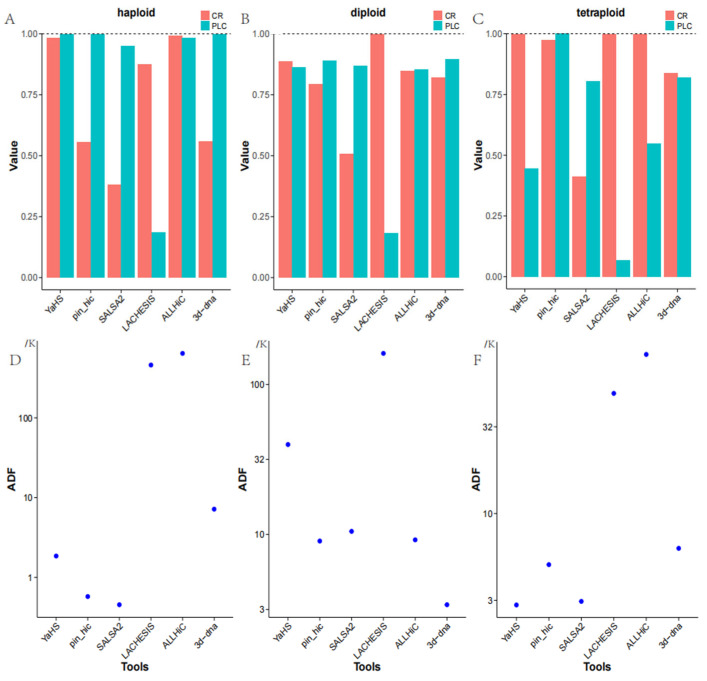
(**A**–**C**) depict the performance of six tools in haploid, diploid, and tetraploid scenarios, encompassing metrics such as Complete Rate (CR) and the mean proportion of the largest category (PLC). (**D**–**F**) illustrate the average distance difference (ADF) of these six tools in haploid, diploid, and tetraploid scenarios, respectively.

In the context of haploid genome assembly, ALLHiC achieved the highest level of completeness at 99.26%, closely followed by YaHS with a completeness of 98.26%. Both of these tools significantly outperformed other alternatives. LACHESIS demonstrated reasonable completeness of 87.54%, whereas pin_hic and 3d-DNA attained completeness rates of only 55.49% and 55.83%, respectively. SALSA2 exhibited the lowest completeness, with a rate of 38.13%. By amalgamating the data presented in Figure 1A and Table 1, we can extract not only the previously mentioned information but also the additional particulars. From a correctness standpoint, as denoted by the PLC metric, YaHS, pin_hic, and 3d-DNA all attained correctness rates exceeding 99.8%. ALLHiC demonstrated a correctness rate of 98.14%, while SALSA2 exhibited a correctness rate of 94.96%. Within the array of tools evaluated, it is noteworthy that LACHESIS exhibited a significantly lower level of correctness, with a corresponding value of 18.63%. As previously mentioned, the majority of these tools demonstrated robust performance in the context of contig grouping, effectively and accurately assigning contigs to their respective groups, except for LACHESIS. Finally, when evaluating the ADF metric to assess the relative ordering of contigs within chromosomes, SALSA2 demonstrated the most superior performance. According to Figure 1D, it is evident that pin_hic also displayed a commendable performance in this aspect. YaHS and 3d-DNA exhibited moderate performance, whereas LACHESIS and ALLHiC displayed relatively suboptimal results. These findings offer valuable insights into the completeness and accuracy of genome assemblies when employing diverse scaffolding tools for haploid genomes.

### 3.2. Diploid Genome

In the context of a diploid genome assembly, LACHESIS demonstrated notably higher completeness, achieving a rate of 99.78%. Conversely, pin_hic and 3d-DNA exhibited comparable levels of completeness, with rates of 79.27% and 82.12%, respectively. Within this context, ALLHiC outperformed both pin_hic and 3d-DNA, achieving a completeness rate of 84.65%, while YaHS surpassed all other tools with the highest completeness rate of 88.57%. SALSA2 exhibited the lowest level of completeness in this scenario, with a rate of only 50.84%. Combining Figure 1B with Table 1, in addition to the information mentioned earlier, we can also glean the following insights. According to the PLC metric, LACHESIS exhibited a notably diminished level of correctness in the context of contig grouping, with a correctness rate of just 18.15%. Conversely, the remaining five software alternatives demonstrated relatively uniform correctness in this aspect, spanning from 85% to 89%, signifying that most tools performed proficiently in terms of contig grouping. Based on Figure 1E, it is evident that, when evaluating the relative ordering of contigs within chromosomes using the ADF metric, 3d-DNA demonstrated the most superior performance. The performance of pin_hic, SALSA2, and ALLHiC exhibited relative similarity, with pin_hic being the top performer among them, followed by ALLHiC and then SALSA2. YaHS displayed comparatively inferior performance, while LACHESIS performed the least favorably. These findings offer valuable insights into the completeness, correctness, and contig ordering performance of various scaffolding tools for diploid genome assemblies.

### 3.3. Tetraploid Genome

For a tetraploid genome assembly, in terms of completeness, YaHS, LACHESIS, and ALLHiC showcased the highest levels of completeness, all surpassing 99.8%. pin_hic also demonstrated commendable performance, achieving a completeness rate of 97.31%, while 3d-DNA’s completeness stood at 83.70%. SALSA2 exhibited the lowest level of completeness, at just 41.35%. Combining Figure 1C with Table 1, in addition to the information mentioned earlier, we can also discern the following insights. When evaluating contig grouping correctness, pin_hic exhibited the most outstanding performance, attaining a correctness rate of 99.98%. SALSA2 and 3d-DNA displayed comparable correctness rates at 80.52% and 82.06%, respectively. YaHS and ALLHiC registered relatively lower correctness rates, at 44.53% and 54.81%, respectively. LACHESIS, on the other hand, exhibited the least favorable performance among the six tools, with a correctness rate of only 6.73%. Based on Figure 1F, it is evident that, according to the ADF metric, YaHS demonstrated the highest level of correctness in linking contigs within groups. SALSA2’s performance closely approached that of YaHS and can be considered favorable. pin_hic exhibited above-average correctness in this respect, with 3d-DNA closely trailing. LACHESIS displayed inferior performance, whereas ALLHiC exhibited the lowest correctness in this regard. These findings offer valuable insights into the completeness, correctness, and contig ordering performance of various scaffolding tools for tetraploid genome assemblies.

### 3.4. Summary

In summary, with respect to haploid genome scaffolding, YaHS showcases the most robust overall performance among the evaluated tools. Although it may not achieve the highest level of accuracy in contig linking when compared to the six tools, it excels notably in terms of completeness and contig grouping correctness. Therefore, in the context of haploid genomes, YaHS emerges as the preeminent tool within this cohort. pin_hic and SALSA2 both demonstrate robust correctness performance across various dimensions. However, it is advisable for researchers to exercise discretion when contemplating the use of these two tools, given their relatively lower levels of completeness, especially if completeness holds significant importance. In the context of diploid genome scaffolding, while LACHESIS excels in terms of completeness, it exhibits notably lower correctness compared to other tools. The other five tools demonstrate uniform and commendable completeness performance. Nevertheless, 3d-DNA distinguishes itself by significantly surpassing the others in terms of contig linking correctness. Hence, for diploid genomes, 3d-DNA is regarded as the top-performing tool, as it upholds a high level of correctness while simultaneously ensuring fundamental completeness. In the context of tetraploid genome scaffolding, pin_hic showcases exceptional completeness and achieves a commendable performance in linking contigs within chromosomes. As a result, pin_hic is deemed the top-performing tool overall. Moreover, 3d-DNA demonstrates performance just slightly below that of pin_hic but outperforms the remaining tools. In contrast, YaHS excels in completeness but lags in terms of contig grouping correctness [15].

## 4. Conclusions

In this research, we conducted an assessment of scaffolding tools that rely on Hi-C data, encompassing genomes with distinct complexities, including haploid, diploid, and polyploid levels. Our study incorporated HiFi data, consisting of both simulated and authentic datasets. We initiated the assembly process using hifiasm and subsequently employed several scaffolding tools to achieve chromosome-scale assembly. Our evaluations revealed that some scaffolding tools exhibit superior completeness while others excel in correctness. Various tools are associated with distinct design biases, which pose a challenge for researchers. In the absence of a reference genome, determining the most suitable tool for a specific dataset can be a daunting task. Based on our findings, for haploid genomes, the performance of most tools is generally on par, with the exception of YaHS. However, when it comes to diploid genomes, only 3d-DNA demonstrates strong performance, while other tools exhibit a lower degree of accuracy. Concerning tetraploid genomes, pin_hic stands out as the top performer, while other tools yield less favorable outcomes.

## Figures and Tables

**Table 1:** The performance of six tools on different genomes.

Tools	Haploid		Diploid		Tetraploid	
	CR	PLC	CR	PLC	CR	PLC
YaHS	0.9826	0.9985	0.8857	0.8618	0.9996	0.4453
pin_hic	0.5549	0.9995	0.7927	0.8905	0.9731	0.9998
SALSA2	0.3813	0.9496	0.5084	0.8680	0.4135	0.8052
LACHESIS	0.8754	0.1863	0.9978	0.1815	0.9996	0.0673
ALLHiC	0.9926	0.9814	0.8465	0.8546	0.9982	0.5482
3d-DNA	0.5583	0.9995	0.8212	0.8947	0.8370	0.8206
